# Epigenetic regulation of tumor immunity

**DOI:** 10.1172/JCI178540

**Published:** 2024-06-17

**Authors:** Lizhi Pang, Fei Zhou, Yang Liu, Heba Ali, Fatima Khan, Amy B. Heimberger, Peiwen Chen

**Affiliations:** Department of Neurological Surgery, Feinberg School of Medicine, Northwestern University, Chicago, Illinois, USA.

## Abstract

Although cancer has long been considered a genetic disease, increasing evidence shows that epigenetic aberrations play a crucial role in affecting tumor biology and therapeutic response. The dysregulated epigenome in cancer cells reprograms the immune landscape within the tumor microenvironment, thereby hindering antitumor immunity, promoting tumor progression, and inducing immunotherapy resistance. Targeting epigenetically mediated tumor-immune crosstalk is an emerging strategy to inhibit tumor progression and circumvent the limitations of current immunotherapies, including immune checkpoint inhibitors. In this Review, we discuss the mechanisms by which epigenetic aberrations regulate tumor-immune interactions and how epigenetically targeted therapies inhibit tumor progression and synergize with immunotherapy.

## Introduction

Tumor microenvironment (TME) heterogeneity is recognized as a cancer hallmark (1–4). Studies using mass cytometry, single-cell RNA-Seq (scRNA-Seq), and spatial transcriptomics/proteomics revealed a dynamic and heterogeneous TME, consisting of supporting extracellular matrix, T cells, and various myeloid cells, including macrophages, myeloid-derived suppressor cells (MDSCs), and neutrophils (5–7). T cells (e.g., CD8^+^ cells) play an important antitumor role across cancer types by inducing granzyme- and perforin-mediated apoptosis. Myeloid cells are usually polarized toward a protumor and immunosuppressive phenotype in response to specific stimuli in the TME (8), thus promoting tumor progression, suppressing T cell–mediated antitumor immunity, and facilitating cancer cell immune escape. Genetic modification, metabolic reprogramming, and epigenetic alteration are the key factors that determine the TME landscape during tumor progression (9–11).

Compared with nonmalignant cells, cancer cells exhibit distinct epigenetic features with regard to histone posttranslational modification, DNA methylation, and RNA modification (1, 12, 13). These epigenetic changes control tumor growth by affecting the biology of oncogenes and/or tumor suppressor genes (12, 14). In addition to these cell-intrinsic effects, cancer cell epigenetic alterations induce immunotherapy resistance by regulating distinct immune cell populations in the TME (15, 16). Recent studies using scRNA-Seq and single-cell sequencing assay for transposase-accessible chromatin sequencing (scATAC-Seq) revealed the association between immune cell composition and chromatin accessibility in cancer cells (17, 18). Notably, cancer cell epigenetic plasticity highly associates with genes located in open chromatin regions that are essential for intercellular communications (19). Epigenetic enzymes and transcriptional mediators in cancer cells can regulate the expression of genes that encode ligands, receptors, and cytokines responsible for immune cell differentiation, migration, and activation (1, 15, 19, 20).

Once infiltrating into the TME, epigenetic reprogramming confers a fitness advantage for these immune cells during tumor progression (21, 22). Certain therapies (e.g., immunotherapy) are also strong stimuli for histone and RNA modifications in tumor-associated immune cells (23–25). Targeting the epigenetic enzymes and factors might potentiate antitumor immunity and synergize with immunotherapies, including immune checkpoint inhibitors (ICIs) (16, 26–28). Given the critical role of immune cells in cancer development (1, 29), understanding the mechanism of epigenetically mediated tumor-immune symbiosis may provide new insights into the discovery of new therapeutic targets. In this Review, we explore the mechanisms for how epigenetic changes in cancer cells affect immune cell biology and how the aberrant epigenome of immune cells affects tumor progression. We also highlight the therapeutic potential of targeting epigenetically dependent tumor-immune communication and its influence on antitumor efficiency of ICI therapy.

## Epigenetic modulation in cancer

Depending on the targets, epigenetic modulation can be classified as chromatin modification, DNA methylation, and RNA modification. Chromatin remodeling is driven by various histone-modifying enzymes, such as histone acetyltransferase, histone deacetylase (HDAC), histone methyltransferase, and histone demethylase (30). These enzymes play an essential role in remodeling chromatin toward an active or repressive state that is accessible or inaccessible to other regulatory factors (12). DNA methylation is catalyzed by DNA methyltransferases (DNMTs) that introduce cytosine at position C5 in CpG dinucleotides. Hypermethylation of pericentromeric heterochromatin and CpG islands in cancer cells contributes to genomic instability and transcription of tumor suppressor genes, allowing cancer cells to better adapt to the TME (31). RNA modification could rapidly rewire the transcriptome and proteome of cancer cells and immune cells in the TME. m^6^A is the most common type of RNA modification that can affect oncogenic networks and tumor immunogenicity (32, 33). Mechanistically, m^6^A modification is regulated by three types of factors, including methyltransferases (writers, e.g., METTL3 and METTL4), demethylases (erasers, e.g., FTO and ALKBH5), and m^6^A binding proteins (readers, e.g., YTHDC1 and YTHDF1). In the nucleus, a writer complex installs m^6^A, a process that can be removed by erasers. The m^6^A nuclear readers recognize m^6^A-modified RNAs, thus regulating mRNA splicing and other nuclear processes. Once mRNAs translocate to cytoplasm from nucleus, m^6^A binds to cytosolic readers, regulating mRNA stability and cancer progression (34).

## Cancer cell epigenetic reprogramming regulates TME biology

Although many patients with cancer benefit from ICIs, their antitumor efficacy can be limited by the immunosuppressive TME (1). Insufficient CD8^+^ T cell infiltration, low PD-L1 expression in cancer cells, and downregulation of major histocompatibility class I (MHC-I) antigen processing and presentation result in a “cold” (immunological ignorance) TME that typically does not respond to ICI therapy (16, 35, 36). These cold tumor properties are associated with epigenetic alterations in cancer cells (27, 37–39). Specifically, these epigenetic alterations suppress the proliferation and activation of T cells and promote the infiltration and immunosuppressive activation of macrophages, MDSCs, and neutrophils by upregulating the expression and secretion of various cytokines and factors (40–42), resulting in immunosuppression and tumor progression in a context-dependent manner (40, 43).

### Cancer cell epigenetics affect T cell biology.

**Figure 1:**
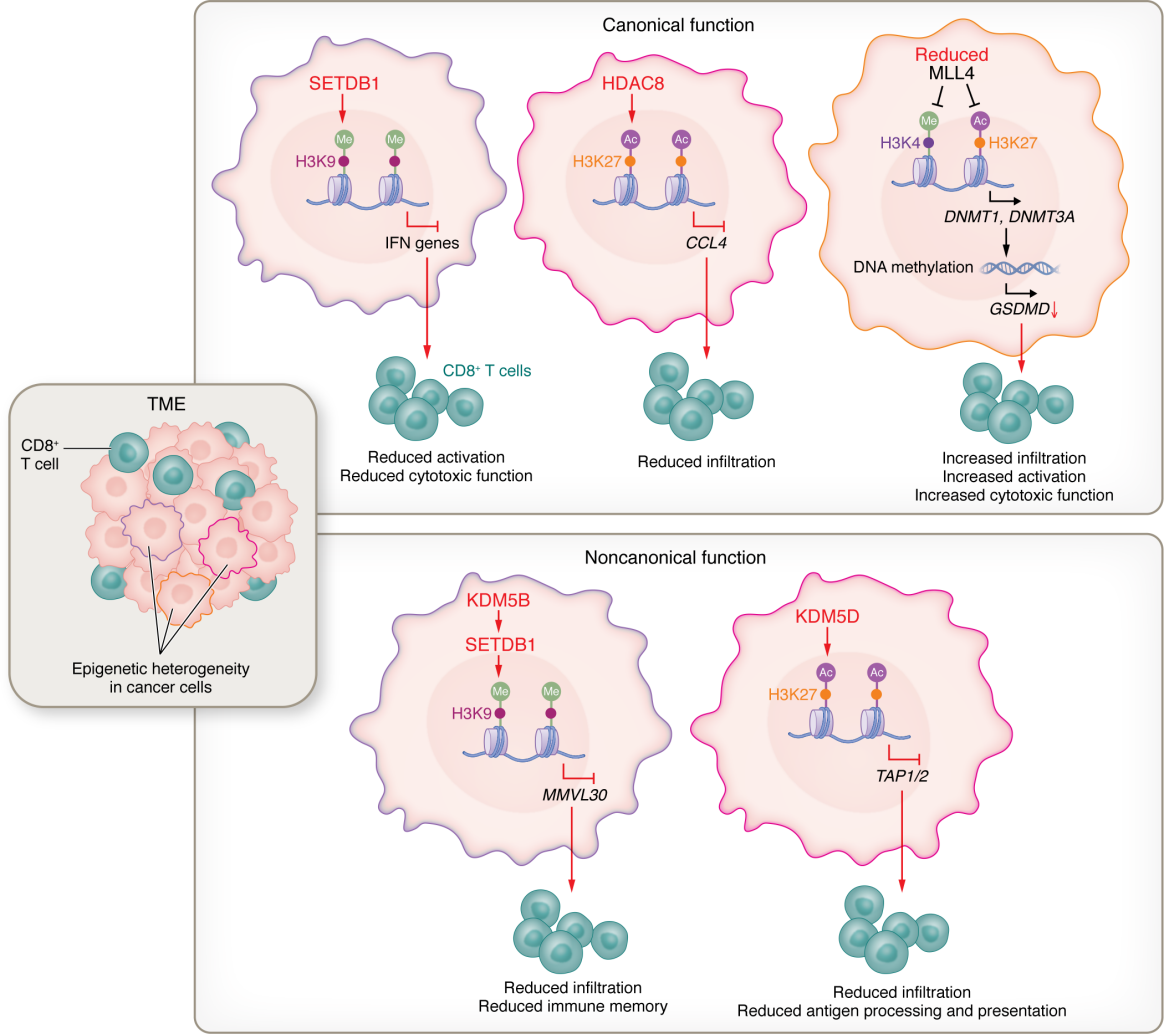
Epigenetic modulations in cancer cells regulate T cell biology. Under selective pressure in the tumor microenvironment (TME), cancer cells exhibit high epigenetic heterogeneity. Aberrant expression of epigenetic enzymes (e.g., SETDB1, HDAC8, and MML4) in cancer cells regulate the expression of immunomodulatory genes (e.g., *IFN* genes, *CCL4*, and *GSDMD*) by catalyzing their classical substrates (e.g., H3K9, H3K27, and H3K4), which, in turn, affect the infiltration, activation, and cytotoxic function of T cells in the TME. Additionally, epigenetic regulators (e.g., KDM5B and KDM5D) in cancer cells also affect T cell antitumor immunity through noncanonical functions. Cancer cells take advantage of these epigenetic modulations to avoid CD8^+^ T cell surveillance, resulting in adaptive clonal expansion. Ac, acetyl group; CCL4, chemokine ligands 4; DNMT1/3a, DNA methyltransferases1/3a; GSDMD, gasdermin D; HDAC8, histone deacetylase 8; KDM5B/D, lysine demethylase 5B/D; Me, methyl group; MLL4, mixed-lineage leukemia 4; MMVL30, virus-like 30S; SETDB1, SET domain bifurcated histone lysine methyltransferase 1; TAP1/2, transporter associated with antigen processing 1/2.

Cancer cells are more easily adapted to the dynamic TME than normal cells because of their epigenetic instability (12, 14, 15, 44). Increasing evidence shows that cancer cell amplification of epigenetic regulators (e.g., H3K36 methyltransferase NSD1, H3K9 methyltransferase SETDB1, histone deacetylase HDAC8, and H3K4 mono-methyltransferase MLL4) is associated with immune modulation (26, 37, 45, 46). These histone-modifying enzymes profoundly regulate cancer cell biology to reshape the immune landscape. In context of cancer cell and T cell crosstalk, SETDB1 mediates intrinsic immunogenicity of cancer cells by catalyzing H3K9 trimethylation and repressing IFN genes (45); HDAC8 in hepatocellular carcinoma cells inhibits CD8^+^ T cell infiltration by reducing H3K27 acetylation and silencing chemokine gene *CCL4* (26); and MLL3 and MLL4 ablation in cancer cells promotes CD8^+^ T cell activation and cytotoxicity by decreasing H3K4me1 and H3K27ac marks and inhibiting *GSDMD* expression (46). Together, cancer cells may take advantage of epigenetics to avoid T cell–mediated immune surveillance (Figure 1).

In contrast to genetic alterations, chromatin-modifying enzymes can target a wide range of domains through thousands of discrete accessible chromatin regions for regulating T cell infiltration and activation (35, 36, 38, 45, 47). SETDB1 is one of such enzymes that exhibits aberrant activity in cancer cells. Amplification of *SETDB1* is associated with segmental duplication events in repression domains of cancer cells that are enriched for immune gene clusters (e.g., IFN gene, Fcγ receptor gene, and ULBP1/RAET1 family gene clusters). *Setdb1* knockout in lung cancer cells or melanoma cells reduces H3K9me3 marks, where about one-third of segmental duplications are enriched for immune-related genes (45). As such, a discrete single epigenetic modification in cancer cells can impact multiple immune-related processes simultaneously (36, 45). Recent advances with CRISPR screening improve the feasibility of identifying vital epigenetic regulators (e.g., MLL4 and SETDB1) that mediate the interplay between cancer cells and T cells (36, 45, 46, 48). scRNA-Seq has also emerged as a powerful technique for evaluating and validating the effect of epigenetic regulators in shaping the immune landscape (49). With the help of this approach, *SETDB1* has been identified in lung carcinoma cells that can regulate CD8^+^ T cell infiltration and recognition and MHC-I presentation (45).

Epigenetic regulators can also mediate tumor immunity independent of their catalytic activity (Figure 1) (50). Lysine-specific demethylase 5B (KDM5B) is such an example (50). Deletion of *Kdm5b* in melanoma cells extends the survival of tumor-bearing mice and increases CD8^+^ T cell infiltration into the TME. Mechanistically, KDM5B represses endogenous retroelements (e.g., *MMVL30*) by recruiting H3K9 methyltransferase SETDB1. Reexpression of either wild-type KDM5B or catalytically inactive KDM5B in *Kdm5b*^–/–^ melanoma cells rescues this phenotype (50). Similarly, KDM5D, located on the Y chromosome, has been found to be a critical epigenetic regulator for inhibiting MHC-I antigen presentation in male colon cancer through a demethylase-independent manner (51). In metastatic cancer cells, KDM5D interacts with the Sin3-HDAC1/2 complex to escape CD8^+^ T cell–mediated tumor killing by downregulating H3K27ac and superenhancer activity (51). Given that certain epigenetic regulators are specifically encoded by X or Y chromosome genes, or regulated by sex hormones (e.g., androgen/pY-SREBF1/H2A-K130ac signaling), these regulators may dictate sex differences in controlling T cell biology in tumors (51, 52).

### Cancer cell epigenetics regulate macrophage biology.

**Figure 2:**
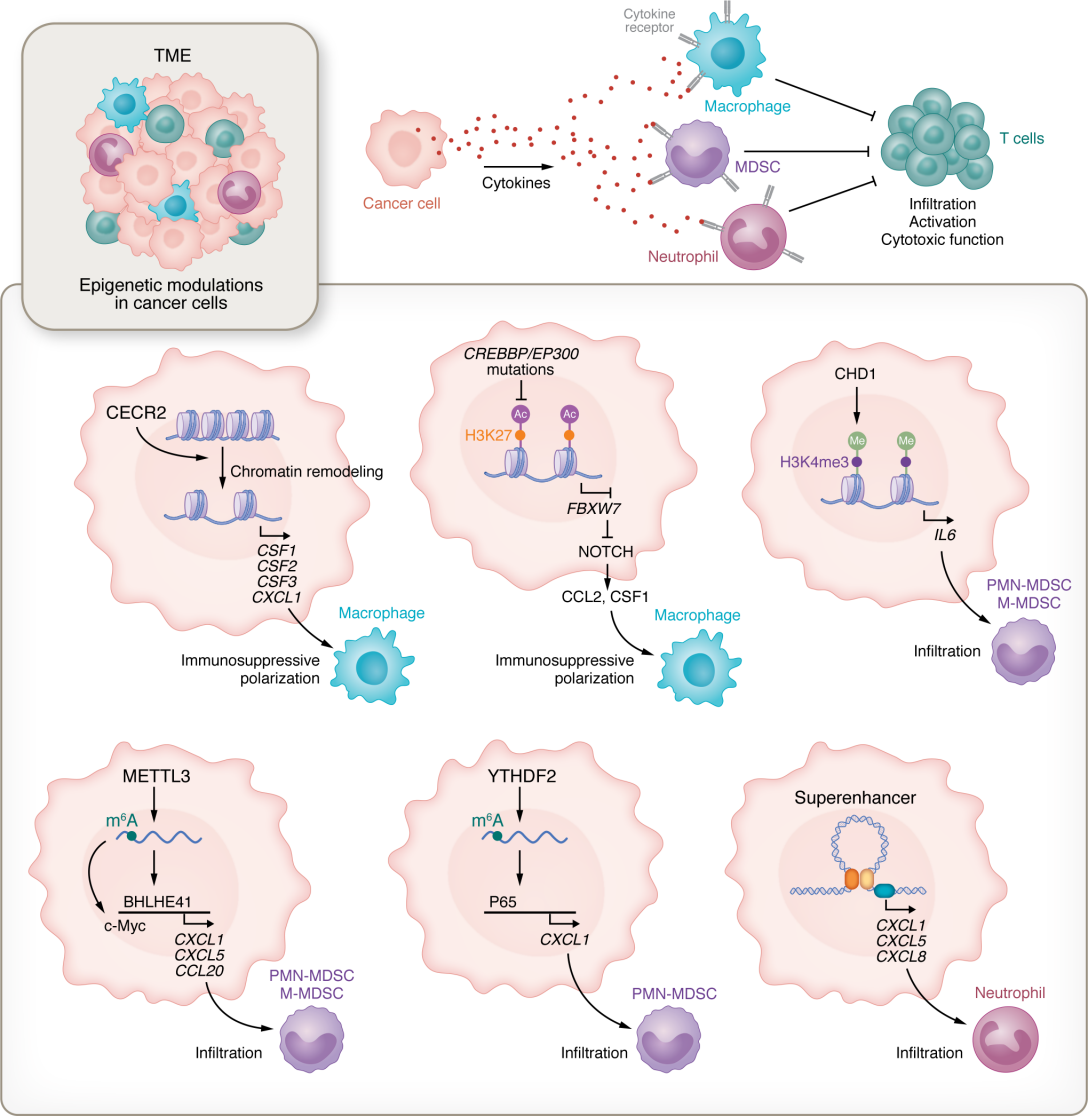
Epigenetic modulations in cancer cells regulate the biology of macrophages, MDSCs, and neutrophils. Epigenetic alterations in cancer cells lead to secretion of various cytokines, chemokines, and factors into the TME. Mechanistically, certain epigenetic modulations (e.g., chromatin remodeling and superenhancer formation) can directly regulate cytokine and chemokine expression. In addition, epigenetic alterations (e.g., histone acetylation and m^6^A mRNA modification) promote cytokine expression through indirect mechanisms, in which other pathways (e.g., NOTCH pathway and NF-κB pathway) control the expression of downstream targeted genes. Cancer cell–secreted cytokines bind to specific cytokine receptors on myeloid cells (e.g., macrophages, MDSCs, and neutrophils), promoting their tumor infiltration and immunosuppressive polarization. Consequently, immunosuppressive myeloid cells inhibit the infiltration, activation, and cytotoxic function of T cells, resulting in immune escape. BHLHE41, basic helix-loop-helix family member e41; CCL20, chemokine ligands 20; CECR2, cat eye syndrome chromosome region candidate 2; CHD1, chromodomain helicase DNA binding protein 1; c-Myc, cellular Myc; CREBBP/EP300, CREB-binding protein and E1A-binding protein P300; CSF1, colony-stimulating factor 1; CXCL1/5/8, C-X-C motif chemokine ligand 1/5/8; m^6^A, N^6^-methyladenosine; MDSC, myeloid-derived suppressor cell; METTL3, methyltransferase like 3; PTEN, phosphatase and tensin homolog; YTHDF2, YTH N6-methyladenosine RNA binding protein 2.

Macrophages are one of the most prominent immune cell populations in tumors across cancer types (53). The density of tumor-associated macrophages (TAMs) is highly correlated with tumor progression and patient survival (53–58). Although many studies have focused on elucidating the relationship between cancer cell genetic alterations (e.g., mutation and deletion of *IDH*, *PTEN*, *TP53*, *NF1*, and *RB1*) and TAM biology (59–62), recent findings suggest that epigenetic reprogramming also regulates the biology (e.g., infiltration and immunosuppressive polarization) of TAMs (20, 57). Mechanistically, epigenetic regulators directly modulate the expression of soluble factors (e.g., cytokines and chemokines), which are responsible for TAM recruitment and immunosuppression (Figure 2). Acetyl-lysine reader CECR2 is such an example that can promote macrophage immunosuppressive polarization by increasing chromatin accessibility and the expression of *CSF1*, *CSF2*, *CSF3*, and *CXCL1* (56). CLOCK has been identified as another epigenetic regulator highly expressed in glioblastoma stem cells, which promotes microglia infiltration and immunosuppressive polarization by transcriptionally upregulating chemokines OLFML3 and LGMN (63, 64).

Another nonnegligible mechanism underpinning TAM regulation is the indirect function of epigenetic regulators on affecting immune regulatory factors. For instance, *CREBBP/EP300* mutations in cancer cells inhibit H3K27 acetylation to downregulate NOTCH suppressor FBXW7, which, in turn, upregulates the expression of CSF1 and CCL2. Consequently, these factors promote macrophage immunosuppressive polarization and tumor growth (Figure 2) (65). Additionally, depletion of m^6^A demethylase ALKBH5 in glioblastoma cells significantly decreases the expression of chemokine CXCL8 under hypoxic conditions. *CXCL8* is not a m^6^A target gene. Instead, ALKBH5 erases m^6^A deposition from the lncRNA NEAT1, accelerating paraspeckle assembly and SFPQ relocation from the CXCL8 promoter (57). Thus, certain epigenetic modulations in cancer cells may hijack other pathways to regulate TAM biology. Given the importance of TAMs in tumor progression, targeting epigenetically driven TAM biology holds great promise for cancer treatment. Experimental findings from cancer mouse models demonstrate that genetic and pharmacologic inhibition of distinct epigenetic factors, such as CECR2, ALKBH5, and EZH2, impairs tumor progression by reducing TAM infiltration and immunosuppressive polarization (27, 56, 57).

### Cancer cell epigenetics regulate MDSC biology.

MDSCs, including polymorphonuclear MDSCs (PMN-MDSCs) and monocytic MDSCs (M-MDSCs), are another critical component of the TME that exhibit potent protumor and immunosuppressive functions. Increasing evidence demonstrates that MDSC biology is modulated by distinct epigenetic regulators in the TME (Figure 2). In *PTEN*-deficient prostate cancer, the expression of epigenetic regulators (e.g., subunit of SWI/SNF chromatin remodeling complex ARID1A and chromatin-remodeling factor CHD1) is positively correlated with MDSC abundance (40, 43, 66, 67). Mechanistically, *PTEN* loss inhibits the degradation of CHD1, which specifically interacts with H3K4me3 to upregulate IL-6, resulting in infiltration of both M-MDSCs and PMN-MDSCs. In contrast to CHD1, ARID1A tends to specifically mediate PMN-MDSC chemotaxis without affecting M-MDSCs. m^6^A appears to be one of the most well-studied epigenetic factors in regulating tumor-MDSC symbiosis (41, 68, 69). Genetic or pharmacologic inhibition of m^6^A writer METTL3 reduces the infiltration of MDSCs in lung cancer (70) and PMN-MDSCs in colorectal cancer (40) by reducing the expression of chemokines CXCL1, CXCL5, and CCL20. METTL3 promotes the translation of transcriptional factor BHLHE41 (40) or increases the stability of *c-Myc* (70), which, in turn, transcriptionally upregulates the expression of these chemokines to trigger M-MDSC and PMN-MDSC infiltration into the TME (Figure 2). In contrast to m^6^A writers, erasers remove m^6^A decoration from RNA. A scRNA-Seq analysis of intrahepatic cholangiocarcinoma tumors demonstrated that knockdown of eraser ALKBH5 upregulates MDSC infiltration (69). However, the opposite effect is observed in colorectal cancer, showing that ALKBH5 promotes the infiltration of PMN-MDSCs and M-MDSCs by directly demethylating AXIN2 messenger RNA (41). These findings suggest that ALKBH5-mediated MDSC infiltration is context and cancer type dependent. Further investigations revealing this context-dependent TME may offer insights into the role and underlying mechanism of ALKBH5 in triggering MDSC infiltration.

In addition to writers and erasers, the expression of m^6^A readers is correlated with tumor immunity (68). Recent studies integrating MeRIP-Seq, RNA-Seq, and Ribo-Seq demonstrated that the NF-κB pathway plays a key role in m^6^A-mediated MDSC accumulation into the TME (25, 55, 68, 71, 72). More specifically, NF-κB subunit p65/Rela is the direct target of YTHDF1 that upregulates chemokine CXCL1 expression in colorectal cancer cells, promoting the infiltration of PMN-MDSCs into the TME (68). The NF-κB pathway may serve as a central hub connecting other epigenetic signals with MDSC chemotaxis (43, 73). For example, ARID1A can induce a positive feedback loop with the NF-κB pathway, resulting in further chemokine expression and PMN-MDSC infiltration (25, 43). Targeting these epigenetic regulators and the downstream signaling of NF-κB pathway has been shown to reduce tumor growth and MDSC infiltration (25, 38, 43, 68).

### Cancer cell epigenetics regulate neutrophil biology.

Neutrophils are the most abundant innate immune cell populations in the circulatory system that can drive immunosuppression. Although neutrophils share many similarities with their immature counterparts PMN-MDSCs (70), they may respond to different cancer cell epigenetic signals. For instance, IDH1 mutation epigenetically upregulates G-CSF expression in glioma cells by increasing H3K4me3 marks on the *CSF3* promoter region, resulting in infiltration of neutrophils, but not PMN-MDSCs (74). In renal cancer cells, epigenetic remodeling promotes the infiltration of mature neutrophils from bone marrow into the TME without affecting myelopoiesis or immature PMN-MDSCs (75). In contrast to tumor-MDSC symbiosis that predominantly contributes to tumor growth (40, 41, 43), recent findings highlight the critical role of epigenetic modulation in neutrophil-induced cancer metastasis (42, 75, 76). DNA methylation and superenhancer (SE) formation in renal cancer cells are required for chemokine (e.g., CXCL8) expression to increase neutrophil infiltration (Figure 2). Inhibition of SE-driven chemokine transcription with BET inhibitor in murine tumor models reduces lung metastasis and prolongs survival in a neutrophil-dependent manner (75). Likewise, histone methyltransferase EZH2 and histone H3K36 trimethyltransferase SETD2 have been reported as vital epigenetic factors regulating neutrophil infiltration during cancer metastasis (42, 77). Unlike its enzymatic function in primary tumors (76), EZH2 promotes melanoma and breast cancer brain metastasis independent of its methyltransferase activity but relying on neutrophils (77). Specifically, tyrosine kinase Src phosphorylates EZH2 at Y696 in brain metastatic cancer cells, which upregulates G-CSF to increase the infiltration of PD-L1^+^ neutrophils, inhibiting CD8^+^ T cell proliferation and promoting metastasis (77, 78). Together, these findings highlight that cancer cell epigenetics plays an important role in regulating neutrophil infiltration and activation in the TME.

## Immune cell epigenetic remodeling affects tumor malignancy

**Figure 3:**
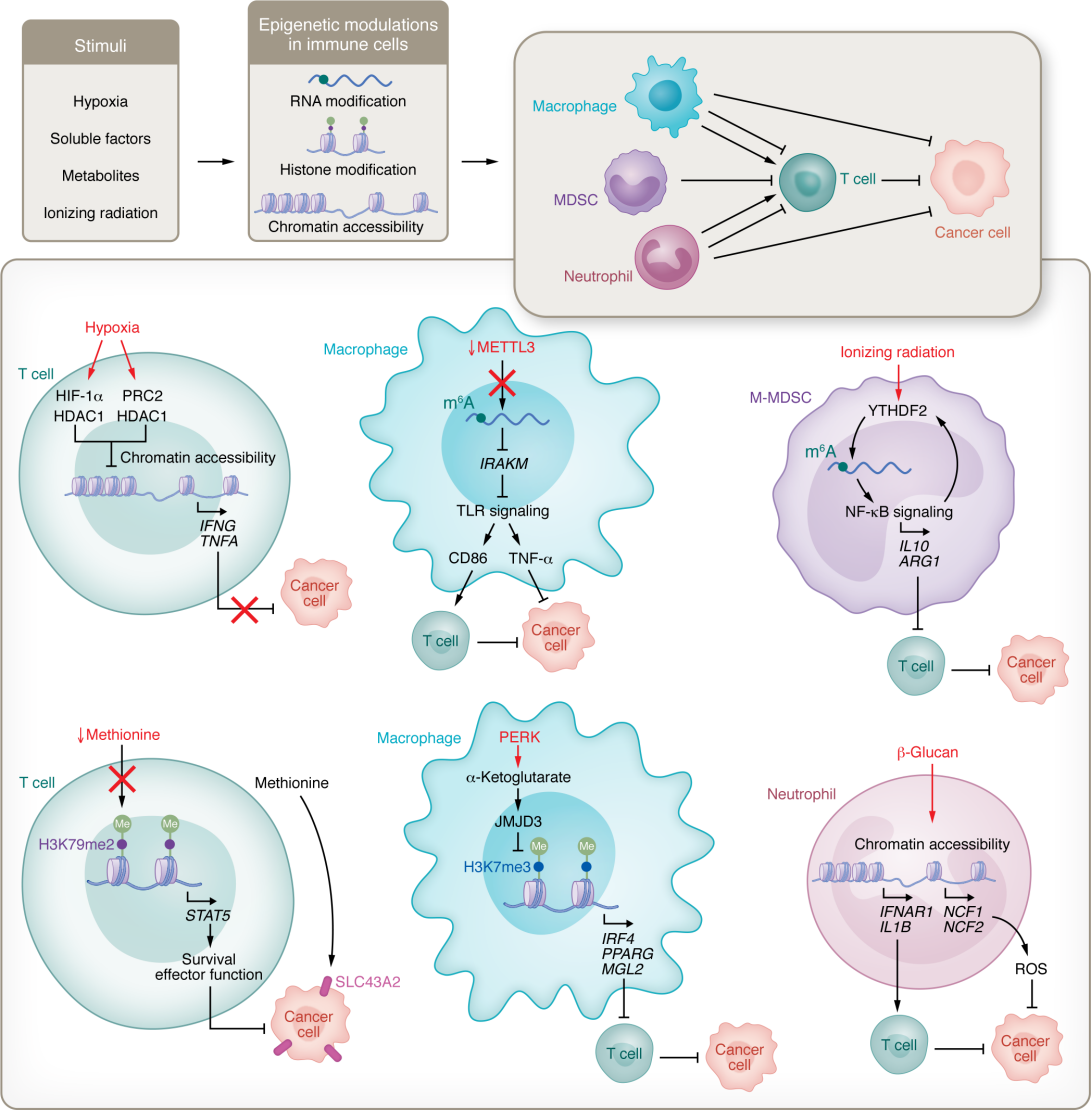
Epigenetic modulations in immune cells regulate cancer cell biology. Various stimuli (e.g., hypoxia, soluble factors, metabolites, and therapeutic interventions) trigger epigenetic alterations in tumor-infiltrating immune cells. These global epigenetic changes upregulate the expression of cytokines or functional molecules (e.g., IFN-γ and TNF-α) in immune cells directly suppressing cancer cell growth. Additional epigenomic changes in innate immune cells regulate cancer cell biology through modulating T cell function. Epigenetic modifications in macrophages, MDSCs, and neutrophils are essential for regulating the expression of genes that encode immunosuppressive factors (e.g., *IL10* and *ARG1*) or proinflammatory cytokines (e.g., *IL1B*). These innate immune cell–derived molecules further induce T cell activation or dampen T cell antitumor function. ARG1, arginase 1; HIF-1α, hypoxia-inducible factor α; IFNAR1, IFN α and β receptor subunit 1; IRAKM, IL-1 receptor–associated kinase 3; IRF4, IFN regulatory factor 4; MGL2, macrophage galactose N-acetyl-galactosamine–specific lectin 2; NFC1/2, neutrophil cytosolic factor 1/2; PERK, protein kinase RNA-like ER kinase; PRC2, polycomb-repressive complex 2; SLC43A2, solute carrier family 43 member 2.

The interplay between cancer cells and immune cells is a two-way street. After infiltrating into the TME that has been engineered by cancer cell signaling (e.g., epigenetic regulators), immune cells reciprocally affect tumor progression and therapy efficiency. Increasing evidence shows that epigenome rewiring in immune cells supports their infiltration, activation, differentiation, antigen presentation, immunosuppression, and exhaustion, resulting in a new TME that drives cancer cell immune escape (21) and facilitates tumor progression (79–81). Here, we discuss how immune cell epigenetics affect tumor progression (Figure 3).

### T cell epigenetics regulate cancer cell biology.

Due to the epigenetic vulnerability and functional dynamics of tumor-infiltrating lymphocytes, targeting epigenetic factors in T cells has emerged as a promising strategy for restoring antitumor immunity (79–81). Depending on the chromatin accessibility, CD8^+^ T cells in the TME can be divided into two epigenetic states: fixed dysfunctional state (marked by high CD38, CD30L, and CD101) and plastic dysfunctional state (marked by high CD5), which are enriched in late-stage and early-stage tumors, respectively (82). Since plastic dysfunctional CD8^+^ T cells are more easily reprogrammed to express immunostimulatory cytokines (82), investigating the mechanisms underlying epigenetic regulation in these T cells might provide vital insight into cancer therapy.

Hypoxia is a hallmark of cancer that drives T cell epigenetic reprogramming (1). The correlation between hypoxia and T cell exhaustion has been shown in many types of solid tumors (79, 80, 83). Hypoxia-inducible factors (HIFs), especially HIF-1α, are the key factors that activate T cell exhaustion program under the hypoxic TME (84, 85). In breast cancer, hypoxia epigenetically downregulates the expression of cytotoxic cytokines (e.g., IFN-γ, TNF-α, and granzyme B) in T cells and promotes them toward an exhaustion-like state without affecting proliferation (80). This inhibitory effect largely depends on the interaction among HIF-1α, PRC2, and HDAC1, which induces chromatin remodeling, resulting in epigenetic suppression of these tumor-killing cytokines (Figure 3) (80). Moreover, exhausted CD8^+^ T cells exhibit an impaired cytotoxic activity, as they harbor fewer active (e.g., H3K27ac) and more repressed chromatin marks (e.g., H3K27me3) (79). This may explain the recent observations that distinct chromatin features are present in T cell progenitors and terminally exhausted tumor-infiltrating T cells under hypoxic conditions (79). Targeting hypoxia-induced chromatin remodeling with HIF-1α inhibitor PX478, deletion of hypoxia-related gene *Ndufs4*, or overexpression of H3K27 histone demethylase KDM6B improves T cell–mediated antitumor efficiency (79, 80).

T cell exhaustion is also attributed to the production of immunosuppressive factors and metabolites in the TME (21, 86–89). Genome-wide CRISPR screening revealed that BAF chromatin remodeling complex is an essential epigenetic regulator for T cell persistence in tumors (81). Depletion of BAF complex member ARID1A or SMARCD2 prevents the terminal exhaustion of T cells and increases memory T cells (81, 90). Given that AR1D1A is also a critical epigenetic factor in cancer cells (38, 91), targeting AR1D1A may gain dual benefits that simultaneously cause cancer cell death and enhance T cell–mediated antitumor activity. Distinct metabolites in the TME can alter the T cell epigenetic landscape (21, 86, 92). Cancer cells uptake exogenous methionine to maintain their high proliferation rate by overexpressing the methionine transporter SLC43A2. Through this mechanism, cancer cells outcompete T cells for methionine, which can maintain H3K79me2 in tumor-infiltrating lymphocytes (21). Due to scarce methionine in the TME, loss of H3K79me2 damages the antitumor immunity of T cells by downregulating the STAT5 pathway (Figure 3) (21). Rewiring metabolic exhaustion of CD8^+^ T cells by supplementation of methionine prevents epigenetic reprogramming and activates T cells for tumor killing (21). Beyond methionine metabolism, m^6^A reader YTHDF1 upregulates the immunosuppressive function of Tregs by maintaining cell hyperglycolysis (86). IDH1 is a metabolic enzyme that catalyzes isocitrate to α-ketoglutarate, a cofactor for histone and DNA demethylases, generating a unique epigenetic landscape to affect antitumor immunity (93–95). On the other hand, the IDH1 mutant can enhance DNA damage response by epigenetically upregulating H3 hypermethylation and ATM signaling (96). As a result, the immune TME is reshaped by DNA damage–induced activation of the cGAS/STING pathway (97–99). Notably, recent findings show that IDH1-mutant cancer cell–derived oncometabolite 2-hydroxyglutarate (2HG) could directly affect CD8^+^ T cell biology in the TME (88, 100). Thus, metabolism may act as a central hub connecting epigenetic alteration and antitumor function in T cells.

### Macrophage epigenetics regulate cancer cell biology.

Given that exhausted T cells and macrophages in the TME are spatiotemporally associated (101), macrophage epigenetic regulators may affect cancer cell biology by regulating T cell functional status. Indeed, TAMs exhibit high MHC-II expression due to chromatin accessibility changes at the early tumor stage (102). These TAMs promote the expansion of Tregs that protect lung cancer cells from adaptive immunity (102). Integration of scRNA-Seq data with cell-cell communication analytical tools and trajectory analysis highlighted the crucial role of m^6^A methylation in TAM-CD8^+^ T cell crosstalk (103). Through this codependency, activated macrophages induce a feedback circuit aligned to protect cancer cells from T cell–mediated tumor killing (101, 103). In addition to this indirect mechanism, CRISPR screening of RNA binding proteins demonstrated that epigenetic alteration in macrophages is critical for tumor progression (104). m^6^A writer METTL3 is a key regulator for macrophage activation (104). METTL3 deficiency in TAMs downregulates m^6^A modification on IRAKM, which, in turn, slows down its degradation and suppresses TLR signaling-mediated TAM activation, inhibiting proinflammatory cytokine expression and promoting tumor progression (Figure 3) (104). YTHDF1 is an essential reader for METTL3-mediated macrophage reprogramming. Depleting the macrophage METTL3-YTHDF1 axis promotes tumor growth and metastasis (55). However, YTHDF1 in dendritic cells (105) and YTHDF2 in macrophages (106) and METTL3 in cancer cells (40, 70) show an opposite effect. Together, these findings highlight the importance of targeting the context-dependent METTL3-YTHDF axis.

In addition to m^6^A modification, metabolism-dependent histone modification regulates TAM immunosuppressive reprogramming. TAMs usually occupy a high lactate microenvironment due to the “Warburg effect” of cancer cells (107). During TAM glycolysis, glucose is converted into pyruvate, which is incorporated into the TCA cycle for citrate production (108). Mitochondria-secreted citrate is further cleaved by ATP-citrate lyase, generating acetyl-coenzyme A for histone acetylation (109). Depleting ATP-citrate lyase decreases the immunosuppressive activity of TAMs (110). Hence, factors controlling metabolic reprogramming may epigenetically regulate TAM activation and its protumor function. An example supporting this hypothesis is the protein kinase RNA-like ER kinase (PERK) signaling cascade, which epigenetically promotes TAM immunosuppression by upregulating serine biosynthesis and mitochondrial function (54). Mechanistically, activated PERK increases α-ketoglutarate production in mitochondria, resulting in upregulation of immunosuppressive genes (e.g., *Irf4*, *Pparg*, and *Mgl2*) by enhancing JMJD3-dependent histone demethylation in TAMs (Figure 3) (54). Finally, DNA methylation enzymes play a fundamental role in macrophage phenotypic changes (111, 112). Tet2 is a typical DNA methylcytosine dioxygenase that catalyzes 5-methylcytosine (5mC) to 5-hydroxymethylcytosine (5hmC) for DNA demethylation (111). Genome-wide 5hmC DNA immunoprecipitation (5hmC-DIP) revealed that Tet2 is required to maintain the low 5mC level at the *Arg1* gene locus (111). Thus, Tet2 supports TAM immunosuppressive polarization to promote tumor progression in melanoma mouse models (111). Together, these findings support the idea that epigenetic reprogramming of TAMs affects tumor progression.

### MDSC epigenetics regulate cancer cell biology.

MDSCs exhibit a potent immunosuppressive and protumor activity by secretion of immunoregulatory factors (25, 113, 114), which are regulated by epigenetic factors, such as H3K27ac regulator CBP/EP300-BRD. Inhibition of CBP/EP300-BRD impairs tumor growth by reducing the migration, differentiation, and function of M-MDSCs and PMN-MDSCs (114). Consistent with these findings, METTL3 is highly enriched in PMN-MDSCs and M-MDSCs of human colon tumors (115). The elevated METTL3-YTHDF1 axis promotes *JAK1* mRNA translation in polysome, upregulating STAT3 signaling in MDSCs to increase the expression of immunosuppressive factors (e.g., IL-6 and IL-10), thus promoting tumor growth by reducing T cell infiltration and activation (115).

Despite these observations, M-MDSCs and PMN-MDSCs may exhibit different roles during tumor progression. Compared with PMN-MDSCs, M-MDSCs are more relevant for promoting lung metastasis in tumor mouse models (116). This finding is consistent with an early study showing that M-MDSCs exhibit stronger immunosuppressive activity than PMN-MDSCs (117). Given that M-MDSCs are sensitive to DNMT inhibitor (118), epigenetic therapy with 5-azacytidine shows a potent antimetastasis activity by downregulating M-MDSC migration, activation, and differentiation (116). Another great example is *Olfr29-ps1*, a m^6^A-regulated pseudogene, that governs MDSC differentiation (119). Upon the epigenetic regulation, *Olfr29-ps1* increases the percentage of M-MDSCs by regulating the *miR-214-3p/*MYD88 signaling axis but decreases the differentiation of PMN-MDSCs (119). Additionally, epigenetic changes induced by therapy, such as ionizing radiation (IR), are critical for MDSC differentiation in the TME. In detail, IR upregulates m^6^A reader YTHDF2 in MDSCs, which, in turn, promotes m^6^A-modified RNA degradation at genes that encode negative regulators of NF-κB signaling. The activated NF-κB/RELA signaling further upregulates YTHDF2 expression in M-MDSCs. This positive circuit between NF-κB and YTHDF2 amplifies the expression of multiple cytokines in M-MDSCs, which promote MDCS infiltration/differentiation and inhibit T cell function to promote tumor growth (Figure 3) (25). Inhibition of YTHDF2 using its specific inhibitor DC-Y13-27 potentiates radiotherapy by reducing M-MDSC infiltration/differentiation in colon cancer and melanoma mouse models (25). However, DC-Y13-27 alone has a limited antitumor effect (25), suggesting that the function of NF-κB-YTHDF2 feedback loop in MDSCs is context dependent.

### Neutrophil epigenetics regulates cancer cell biology.

Neutrophils are a heterogeneous population of innate immune cells in the TME (120, 121). Epigenetic mediators (e.g., METTL3 and KDM6B) are essential for neutrophil development and activation (122–124). By modifying the mRNA of TLR4, m^6^A writer METTL3 promotes neutrophil migration and production of proinflammatory cytokines (e.g., TNF-α, IL-6, and IL-1β). However, it is still unclear how neutrophils respond to METTL3 inhibition in the TME. Myeloid-specific deletion of *Kdm6b* reduces the abundance of neutrophils in glioblastoma tumors (124), indicating that histone demethylation might promote neutrophil infiltration. Infiltrated neutrophils secrete various cytokines to suppress T cell activation and proliferation, thus promoting tumor progression (120). Neutrophils may also exhibit antitumor effects independent of T cell–mediated immunity (125). For example, pretreatment with β-glucan in mice can reprogram circulating neutrophils into an antitumor phenotype that suppresses tumor growth irrespective of adaptive immunity in the host (126). While type I IFN signaling activation is required for this phenotype switch, β-glucan–induced trained immunity increases chromatin accessibility of ROS-producing genes (e.g., *NCF1/2*) in neutrophils (126). Functional studies demonstrated that adoptive transfer of β-glucan–pretreated neutrophils into recipient mice inhibits tumor growth (Figure 3) (126). As epigenetic rewiring of granulopoiesis reprograms neutrophils to an antitumor phenotype (126), it is conceivable that epigenetically modulated neutrophils represents a promising avenue for immunologically cold tumors.

## Targeting epigenetic regulators for cancer immunotherapy

Cancer immunotherapies, including ICIs, have undergone remarkable advancement over the years. However, the TME poses a challenge for developing effective immunotherapies owing to its heterogeneous and immunosuppressive nature (127, 128). Epigenetic reprogramming is critical for governing the formation of such TMEs, resulting in impaired T cell activation and accumulated immunosuppressive cells, including Tregs, TAMs, and MDSCs (56). Given the symbiotic interaction between cancer cells and immune cells, epigenetic reprogramming in each cell component can reshape the TME to affect immunotherapy efficiency (129, 130). Therefore, targeting pivotal epigenetic modulators may change the TME to overcome the limited effects of cancer immunotherapies (131).

DNMT and HDAC inhibitors are the most widely used drugs for cancer treatment, with the potential to improve the antitumor efficiency of immunotherapy (132–134). Decitabine (DAC) is an FDA-approved DNMT inhibitor that has been approved for hematological malignancy treatment (135). Despite DAC’s limited efficacy on solid tumors as a monotherapy, a recent study revealed that DAC significantly enhanced the antitumor effect of anti-PD1 or anti-VISTA in pancreatic ductal adenocarcinoma (136). Similarly, inhibition of HDAC1 synergized with anti-PD1 by preventing T cell exhaustion in breast cancer (80), and treatment with HDAC8 inhibitor PCI-34051 combined with anti-PD-L1 induced a robust antitumor effect in liver cancer (26). Inhibiting EZH2 using DZNep or EPZ can turn the tumor from cold to hot by activating the dsRNA/STING/IFN axis or downregulating the expression of protumor inflammatory cytokine G-CSF, resulting in upregulation of activated CD8^+^ T cells and downregulation of immunosuppressive neutrophils. As a result, EZH2 inhibition synergized with anti-PD1 therapy in prostate and breast cancer (27, 77). Additionally, pharmacologic inhibition of other methyltransferases (e.g., PRMT5, SMYD3, or MLL4 methyltransferase) or specific deletion of these epigenetic regulators in cancer cells has also been found to specifically increase the antitumor efficacy of anti-PD1 therapy across cancer types (46, 137–140). Apart from inhibitors aiming at inhibiting histone modification and DNA methylation, targeting the chromatin remodeling regulator CECR2 using its specific inhibitors (e.g., GNE-886 and NVS-CECR2-1) can effectively enhance antitumor immunity (56). Targeting chromatin remodeling complex ARID1A or its downstream effector NF-κB can also reshape the immunosuppressive TME by inducing IFN expression and reducing PMN-MDSC recruitment, thereby offering benefits to anti-PD1/CTLA-4 for cancer treatment (38, 43). Together, these findings suggest that targeting epigenetics can greatly improve ICI antitumor efficiency (Table 1).

Because T cells can eliminate cancer cells (141), directly targeting the epigenome in tumor-infiltrating CD8^+^ T cells to preserve their effector functions is another strategy being explored in combination with ICIs (Table 1) (81, 142–144). Suv39h1, a histone methyltransferase, has been found to promote T cell exhaustion by downregulating IFN-γ and granzyme B in effector CD8^+^ T cells. Treatment with the Suv39h1 inhibitor ETP-69 combined with anti-PD1 induced an increased proportion of effector CD8^+^ T cells and impaired tumor growth (23). Furthermore, treatment with histone demethylase LSD1 inhibitor GSK2879552 induced a long-lasting response of anti-PD1 in colon cancer by reinvigorating exhausted CD8^+^ T cells in the TME (142). Treatment with KDM6B inhibitor GSK-J4 enhanced the efficacy of anti-PD1 therapy in glioblastoma by decreasing CD14^+^Ly6c2^+^CXCL2^+^VEGFA^+^ monocytic macrophages and increasing CD8^+^GZMB^+^IFN-γ^+^ cytotoxic T cells (124). Moreover, hypoxia-activated epigenetic machinery (e.g., HDAC1) in CD8^+^ T cells suppressed immune effector activity by downregulating IFN-γ. HDAC1 inhibition using its inhibitor entinostat dramatically boosted the antitumor efficiency of anti-PD1 therapy in breast cancer (80). Targeting BATF in Tregs inhibited chromatin accessibility and reduced the expression of CTLA4, ICOS, GITR, and PD-1, suppressing Treg activation and providing a stronger antitumor effect when combined with anti-PD1 therapy (145). Besides targeting aberrant epigenome alterations in T cells, modulators affecting other immune cells such as macrophages and MSDCs can also synergize with ICIs (Table 1). CN133, a novel HDAC inhibitor that impairs the function of PMN-MDSCs by reducing the expression of immunosuppressive enzymes, has been found to markedly improve the antitumor efficacy of anti-PD1 treatment in prostate cancer (146). Considering these epigenetic regulators’ pivotal role in immune cell activation and exhaustion, coinhibiting “epigenetic checkpoints” and classical immune checkpoints (e.g., PD1 or CTLA-4) may achieve a synergistic antitumor effect (27, 80, 147).

In addition to solid tumors, epigenetic drugs (e.g., DNMT inhibitor azacytidine) show clinical benefits in hematologic malignancies, including acute myeloid leukemia (AML) (148, 149). It should be noted that the effectiveness of epigenetic therapies in AML is context dependent. AML cells harboring somatic mutations in *DNMT3A* appear more susceptible to azacytidine treatment (150). Conversely, AML cells expressing high levels of ubiquitin ligase RNF5 show less sensitivity to HDAC inhibitors (151). Given that epigenetic changes in AML cells can modulate cell-intrinsic immune response (152), it is reasonable to develop epigenetic immunotherapies by combining epigenetic drugs with chimeric antigen receptor (CAR) T cell therapies (153). Indeed, preclinical studies showed that treatment with azacytidine increases CD123 expression, resulting in enhanced recognition and elimination of AML cells in response to anti-CD123 CAR T cells (153). Together, these findings highlight that epigenetic immunotherapies may hold great promise for hematologic malignancy treatment.

## Concluding remarks

Immunotherapy, including ICI, has revolutionized current oncology treatment (1, 16, 154). However, a plethora of clinical trials have shown that many patients with cancer ultimately develop resistance to ICIs (155–157). The TME is now recognized as a critical barrier impairing the efficacy of ICI (5, 158, 159). Given the heterogeneity of the TME across cancer types, specifically targeting this context-dependent interplay between the TME and cancer cells is a recognized strategy for sensitizing nonresponder tumors to ICIs (11, 26, 27, 160). In addition to genetic alterations, epigenetic remodeling plays an important role in regulating TME heterogeneity (36, 161, 162). Because cancer cells have a more plastic and unstable epigenome than nonmalignant cells, they can easily adapt to the selection pressures and exhibit a distinct epigenetic state that can further allow them to activate alternative gene regulatory programs (12, 35, 163, 164), including genes for controlling T cell immunity (Figure 1) (51, 138). This theory may explain the significant correlation between epigenetic alterations in cancer cells and T cell dysfunction (79, 165). Additionally, cancer cells that have undergone epigenome alteration release various cytokines and chemokines within the TME, recruiting and activating tumor-associated myeloid cells (e.g., macrophages, MDSCs, and neutrophils). These cells further shape an immunosuppressive TME by limiting the proliferation and function of cytotoxic T cells (Figure 2). Given the heterogeneity and plasticity of the myeloid cells in the TME, future studies are still needed to elucidate the role and underlying mechanism of cancer cell epigenetic change—driven immune landscape in a context-dependent manner.

Furthermore, epigenetic changes in immune cells contribute to tumor progression (22, 145). Unlike cancer cell epigenetics that are mainly attributed to genetic stimuli (166), immune cell epigenetics are primarily triggered by stimuli from the TME, such as hypoxia and cancer cell–derived factors or metabolites (Figure 3) (80, 110, 167, 168). Tumor-associated myeloid cells (e.g., TAMs) exhibit a superior sensitivity to metabolite changes (107, 110, 169, 170) and can switch their phenotypes upon epigenetic reprogramming (54, 107, 110, 111, 169, 171). Thus, targeting the altered epigenetic states of myeloid cells may inhibit tumor growth and improve T cell activity to yield new therapeutic strategies for overcoming ICI resistance (Table 1) (55, 106, 124). Of note, cancer treatments are also able to drive epigenetic modifications in cancer cells and immune cells (12, 24, 25). Myeloid cells, such as MDSCs, take advantage of this mechanism to further impede tumor immunity even in the presence of such treatment (25). Despite these important findings, it is still unclear whether epigenetic regulators affect myeloid cell lineage plasticity and dynamics in the TME and how this connection consequently regulates cancer cell survival and ICI resistance.

Single-cell approaches have emerged as powerful tools to decipher different layers of epigenome in cancer cells and immune cells simultaneously (18, 22, 49, 172). We postulate that the integration of these techniques with CRISPR screening may help identify unforeseen epigenetic elements that are crucial for tumor-immune interplay. Despite the progress, single-cell epigenetic technologies are still limited by low throughput, poor coverage, and lack of multiplexing capabilities (18). Developing epigenetic long-read single-cell sequencing may provide new insights into how epigenomic heterogeneity is related to the codependency between immune cells and cancer cells (173). Given the dynamic epigenomic landscape of immune cells (174–176), we are optimistic that a deeper understanding of mechanisms connecting epigenetic modifications to tumor immunity will advance the development of novel immunotherapies.

## Figures and Tables

**Table 1:**
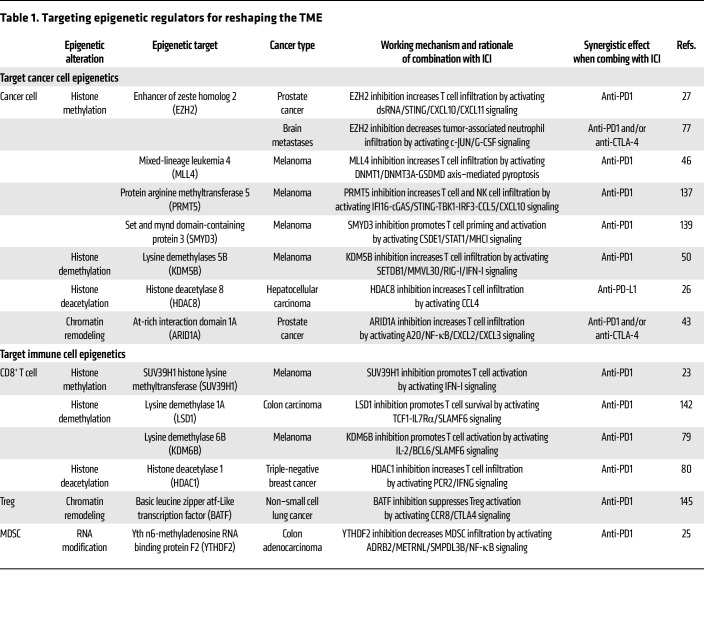
Targeting epigenetic regulators for reshaping the TME

## Version 1 06/17/2024

Electronic publication

## References

[1] Hanahan D (2022). Hallmarks of cancer: new dimensions. Cancer Discov.

[2] de Visser KE, Joyce JA (2023). The evolving tumor microenvironment: From cancer initiation to metastatic outgrowth. Cancer Cell.

[3] Anderson NM, Simon MC (2020). The tumor microenvironment. Curr Biol.

[4] Binnewies M (2018). Understanding the tumor immune microenvironment (TIME) for effective therapy. Nat Med.

[5] Pang L (2022). Pharmacological targeting of the tumor-immune symbiosis in glioblastoma. Trends Pharmacol Sci.

[6] Davis-Marcisak EF (2021). From bench to bedside: single-cell analysis for cancer immunotherapy. Cancer Cell.

[7] Pang L (2023). Circadian regulator CLOCK promotes tumor angiogenesis in glioblastoma. Cell Rep.

[8] Ma R-Y (2022). Macrophage diversity in cancer revisited in the era of single-cell omics. Trends Immunol.

[9] van Vlerken-Ysla L (2023). Functional states of myeloid cells in cancer. Cancer Cell.

[10] Ravi VM (2022). T-cell dysfunction in the glioblastoma microenvironment is mediated by myeloid cells releasing interleukin-10. Nat Commun.

[11] Pang L (2023). Kunitz-type protease inhibitor TFPI2 remodels stemness and immunosuppressive tumor microenvironment in glioblastoma. Nat Immunol.

[12] Flavahan WA (2017). Epigenetic plasticity and the hallmarks of cancer. Science.

[13] Choe J (2018). mRNA circularization by METTL3-eIF3h enhances translation and promotes oncogenesis. Nature.

[14] Sarkies P, Sale JE (2012). Cellular epigenetic stability and cancer. Trends Genet.

[15] Davies A (2023). The transcriptional and epigenetic landscape of cancer cell lineage plasticity. Cancer Discov.

[16] Topper MJ (2020). The emerging role of epigenetic therapeutics in immuno-oncology. Nat Rev Clin Oncol.

[17] Becker WR (2022). Single-cell analyses define a continuum of cell state and composition changes in the malignant transformation of polyps to colorectal cancer. Nat Genet.

[18] Casado-Pelaez M (2022). Single cell cancer epigenetics. Trends Cancer.

[19] Burdziak C (2023). Epigenetic plasticity cooperates with cell-cell interactions to direct pancreatic tumorigenesis. Science.

[20] Gangoso E (2021). Glioblastomas acquire myeloid-affiliated transcriptional programs via epigenetic immunoediting to elicit immune evasion. Cell.

[21] Bian Y (2020). Cancer SLC43A2 alters T cell methionine metabolism and histone methylation. Nature.

[22] Kourtis N (2022). A single-cell map of dynamic chromatin landscapes of immune cells in renal cell carcinoma. Nat Cancer.

[23] Niborski LL (2022). CD8+T cell responsiveness to anti-PD-1 is epigenetically regulated by Suv39h1 in melanomas. Nat Commun.

[24] Pauken KE (2016). Epigenetic stability of exhausted T cells limits durability of reinvigoration by PD-1 blockade. Science.

[25] Wang L (2023). YTHDF2 inhibition potentiates radiotherapy antitumor efficacy. Cancer Cell.

[26] Yang W (2021). A selective HDAC8 inhibitor potentiates antitumor immunity and efficacy of immune checkpoint blockade in hepatocellular carcinoma. Sci Transl Med.

[27] Morel KL (2021). EZH2 inhibition activates a dsRNA-STING-interferon stress axis that potentiates response to PD-1 checkpoint blockade in prostate cancer. Nat Cancer.

[28] Liu Y (2024). Epigenetic regulation of tumor-immune symbiosis in glioma. Trends Mol Med.

[29] Chen P (2021). Cancer stemness meets immunity: from mechanism to therapy. Cell Rep.

[30] Bannister AJ, Kouzarides T (2011). Regulation of chromatin by histone modifications. Cell Res.

[31] Nishiyama A, Nakanishi M (2021). Navigating the DNA methylation landscape of cancer. Trends Genet.

[32] Li X (2022). Targeting the RNA m^6^A modification for cancer immunotherapy. Mol Cancer.

[33] Wei T (2023). Loss of Mettl3 enhances liver tumorigenesis by inducing hepatocyte dedifferentiation and hyperproliferation. Cell Rep.

[34] Murakami S, Jaffrey SR (2022). Hidden codes in mRNA: Control of gene expression by m^6^A. Mol Cell.

[35] Zhou Y (2021). Activation of NF-κB and p300/CBP potentiates cancer chemoimmunotherapy through induction of MHC-I antigen presentation. Proc Natl Acad Sci U S A.

[36] Burr ML (2019). An evolutionarily conserved function of polycomb silences the MHC class I antigen presentation pathway and enables immune evasion in cancer. Cancer Cell.

[37] Li Y (2022). Histone methylation antagonism drives tumor immune evasion in squamous cell carcinomas. Mol Cell.

[38] Li J (2020). Epigenetic driver mutations in ARID1A shape cancer immune phenotype and immunotherapy. J Clin Invest.

[39] Yuan H (2023). Lysine catabolism reprograms tumour immunity through histone crotonylation. Nature.

[40] Chen H (2022). METTL3 inhibits antitumor immunity by targeting m^6^A-BHLHE41-CXCL1/CXCR2 axis to promote colorectal cancer. Gastroenterology.

[41] Zhai J (2023). ALKBH5 drives immune suppression via targeting AXIN2 to promote colorectal cancer and is a target for boosting immunotherapy. Gastroenterology.

[42] Niu N (2023). Tumor cell-intrinsic SETD2 deficiency reprograms neutrophils to foster immune escape in pancreatic tumorigenesis. Adv Sci (Weinh).

[43] Li N (2022). ARID1A loss induces polymorphonuclear myeloid-derived suppressor cell chemotaxis and promotes prostate cancer progression. Nat Commun.

[44] Reik W (2007). Stability and flexibility of epigenetic gene regulation in mammalian development. Nature.

[45] Griffin GK (2021). Epigenetic silencing by SETDB1 suppresses tumour intrinsic immunogenicity. Nature.

[46] Ning H (2022). Enhancer decommissioning by MLL4 ablation elicits dsRNA-interferon signaling and GSDMD-mediated pyroptosis to potentiate anti-tumor immunity. Nat Commun.

[47] Guo H (2023). DNA hypomethylation silences anti-tumor immune genes in early prostate cancer and CTCs. Cell.

[48] Wu Y (2023). Disrupting the phase separation of KAT8-IRF1 diminishes PD-L1 expression and promotes antitumor immunity. Nat Cancer.

[49] Yu Z (2023). Integrative single-cell analysis reveals transcriptional and epigenetic regulatory features of clear cell renal cell carcinoma. Cancer Res.

[50] Zhang S-M (2021). KDM5B promotes immune evasion by recruiting SETDB1 to silence retroelements. Nature.

[51] Li J (2023). Histone demethylase KDM5D upregulation drives sex differences in colon cancer. Nature.

[52] Nguyen T (2023). Histone H2A Lys130 acetylation epigenetically regulates androgen production in prostate cancer. Nat Commun.

[53] Qian B-Z, Pollard JW (2010). Macrophage diversity enhances tumor progression and metastasis. Cell.

[54] Raines LN (2022). PERK is a critical metabolic hub for immunosuppressive function in macrophages. Nat Immunol.

[55] Yin H (2021). RNA m6A methylation orchestrates cancer growth and metastasis via macrophage reprogramming. Nat Commun.

[56] Zhang M (2022). CECR2 drives breast cancer metastasis by promoting NF-κB signaling and macrophage-mediated immune suppression. Sci Transl Med.

[57] Dong F (2021). ALKBH5 facilitates hypoxia-induced paraspeckle assembly and IL8 secretion to generate an immunosuppressive tumor microenvironment. Cancer Res.

[58] Khan F (2024). Lactate dehydrogenase A regulates tumor-macrophage symbiosis to promote glioblastoma progression. Nat Commun.

[59] Pang L (2022). Mechanism and therapeutic potential of tumor-immune symbiosis in glioblastoma. Trends Cancer.

[60] Venteicher AS (2017). Decoupling genetics, lineages, and microenvironment in IDH-mutant gliomas by single-cell RNA-seq. Science.

[61] Wellenstein MD (2019). Loss of p53 triggers WNT-dependent systemic inflammation to drive breast cancer metastasis. Nature.

[62] Chen P (2019). Symbiotic macrophage-glioma cell interactions reveal synthetic lethality in PTEN-null glioma. Cancer Cell.

[63] Xuan W (2022). Circadian regulator CLOCK drives immunosuppression in glioblastoma. Cancer Immunol Res.

[64] Chen P (2020). Circadian regulator CLOCK recruits immune-suppressive microglia into the GBM tumor microenvironment. Cancer Discov.

[65] Huang Y-H (2021). CREBBP/EP300 mutations promoted tumor progression in diffuse large B-cell lymphoma through altering tumor-associated macrophage polarization via FBXW7-NOTCH-CCL2/CSF1 axis. Sig Transduct Target Ther.

[66] Ni H (2020). Connecting METTL3 and intratumoural CD33^+^ MDSCs in predicting clinical outcome in cervical cancer. J Transl Med.

[67] Zhao D (2020). Chromatin regulator CHD1 remodels the immunosuppressive tumor microenvironment in PTEN-deficient prostate cancer. Cancer Discov.

[68] Bao Y (2023). Targeting m^6^A reader YTHDF1 augments antitumour immunity and boosts anti-PD-1 efficacy in colorectal cancer. Gut.

[69] Qiu X (2021). M^6^A demethylase ALKBH5 regulates PD-L1 expression and tumor immunoenvironment in intrahepatic cholangiocarcinoma. Cancer Res.

[70] Yu H (2023). Targeting METTL3 reprograms the tumor microenvironment to improve cancer immunotherapy. Cell Chem Biol.

[71] Qu J (2022). RNA demethylase ALKBH5 promotes tumorigenesis in multiple myeloma via TRAF1-mediated activation of NF-κB and MAPK signaling pathways. Oncogene.

[72] He J (2021). METTL3 restrains papillary thyroid cancer progression via m^6^A/c-Rel/IL-8-mediated neutrophil infiltration. Mol Ther.

[73] Porta C (2020). Tumor-derived prostaglandin E2 promotes p50 NF-κB-dependent differentiation of monocytic MDSCs. Cancer Res.

[74] Alghamri MS (2021). G-CSF secreted by mutant IDH1 glioma stem cells abolishes myeloid cell immunosuppression and enhances the efficacy of immunotherapy. Sci Adv.

[75] Nishida J (2020). Epigenetic remodelling shapes inflammatory renal cancer and neutrophil-dependent metastasis. Nat Cell Biol.

[76] Chibaya L (2023). EZH2 inhibition remodels the inflammatory senescence-associated secretory phenotype to potentiate pancreatic cancer immune surveillance. Nat Cancer.

[77] Zhang L (2020). Blocking immunosuppressive neutrophils deters pY696-EZH2-driven brain metastases. Sci Transl Med.

[78] Wang T (2017). Tumour-activated neutrophils in gastric cancer foster immune suppression and disease progression through GM-CSF-PD-L1 pathway. Gut.

[79] Ford BR (2022). Tumor microenvironmental signals reshape chromatin landscapes to limit the functional potential of exhausted T cells. Sci Immunol.

[80] Ma S (2022). Hypoxia induces HIF1α-dependent epigenetic vulnerability in triple negative breast cancer to confer immune effector dysfunction and resistance to anti-PD-1 immunotherapy. Nat Commun.

[81] Belk JA (2022). Genome-wide CRISPR screens of T cell exhaustion identify chromatin remodeling factors that limit T cell persistence. Cancer Cell.

[82] Philip M (2017). Chromatin states define tumour-specific T cell dysfunction and reprogramming. Nature.

[83] Cunha PP (2023). Oxygen levels at the time of activation determine T cell persistence and immunotherapeutic efficacy. Elife.

[84] Doedens AL (2013). Hypoxia-inducible factors enhance the effector responses of CD8(+) T cells to persistent antigen. Nat Immunol.

[85] Franco F (2020). Metabolic and epigenetic regulation of T-cell exhaustion. Nat Metab.

[86] Wang A (2023). USP47 inhibits m6A-dependent c-Myc translation to maintain regulatory T cell metabolic and functional homeostasis. J Clin Invest.

[87] Chapman NM, Chi H (2022). Metabolic adaptation of lymphocytes in immunity and disease. Immunity.

[88] Notarangelo G (2022). Oncometabolite d-2HG alters T cell metabolism to impair CD8^+^ T cell function. Science.

[89] Reina-Campos M (2023). Metabolic programs of T cell tissue residency empower tumour immunity. Nature.

[90] Guo A (2022). cBAF complex components and MYC cooperate early in CD8^+^ T cell fate. Nature.

[91] Zhang T (2022). ADAR1 masks the cancer immunotherapeutic promise of ZBP1-driven necroptosis. Nature.

[92] Koss B (2020). Epigenetic control of *Cdkn2a.Arf* protects tumor-infiltrating lymphocytes from metabolic exhaustion. Cancer Res.

[93] McClellan BL (2023). Impact of epigenetic reprogramming on antitumor immune responses in glioma. J Clin Invest.

[94] Flavahan WA (2016). Insulator dysfunction and oncogene activation in IDH mutant gliomas. Nature.

[95] Amankulor NM (2017). Mutant IDH1 regulates the tumor-associated immune system in gliomas. Genes Dev.

[96] Núñez FJ (2019). IDH1-R132H acts as a tumor suppressor in glioma via epigenetic up-regulation of the DNA damage response. Sci Transl Med.

[97] Mackenzie KJ (2017). cGAS surveillance of micronuclei links genome instability to innate immunity. Nature.

[98] Leuzzi G (2024). SMARCAL1 is a dual regulator of innate immune signaling and PD-L1 expression that promotes tumor immune evasion. Cell.

[99] Kwon J, Bakhoum SF (2020). The cytosolic DNA-sensing cGAS-STING pathway in cancer. Cancer Discov.

[100] Foskolou IP (2023). The two enantiomers of 2-hydroxyglutarate differentially regulate cytotoxic T cell function. Cell Rep.

[101] Kersten K (2022). Spatiotemporal co-dependency between macrophages and exhausted CD8^+^ T cells in cancer. Cancer Cell.

[102] Casanova-Acebes M (2021). Tissue-resident macrophages provide a pro-tumorigenic niche to early NSCLC cells. Nature.

[103] Dong L (2021). The loss of RNA N^6^-adenosine methyltransferase Mettl14 in tumor-associated macrophages promotes CD8^+^ T cell dysfunction and tumor growth. Cancer Cell.

[104] Tong J (2021). Pooled CRISPR screening identifies m^6^A as a positive regulator of macrophage activation. Sci Adv.

[105] Han D (2019). Anti-tumour immunity controlled through mRNA m^6^A methylation and YTHDF1 in dendritic cells. Nature.

[106] Ma S (2023). YTHDF2 orchestrates tumor-associated macrophage reprogramming and controls antitumor immunity through CD8^+^ T cells. Nat Immunol.

[107] Zhang D (2019). Metabolic regulation of gene expression by histone lactylation. Nature.

[108] Bricker DK (2012). A mitochondrial pyruvate carrier required for pyruvate uptake in yeast, Drosophila, and humans. Science.

[109] Wellen KE (2009). ATP-citrate lyase links cellular metabolism to histone acetylation. Science.

[110] Noe JT (2021). Lactate supports a metabolic-epigenetic link in macrophage polarization. Sci Adv.

[111] Pan W (2017). The DNA methylcytosine dioxygenase Tet2 sustains immunosuppressive function of tumor-infiltrating myeloid cells to promote melanoma progression. Immunity.

[112] Wang Y-C (2018). USP24 induces IL-6 in tumor-associated microenvironment by stabilizing p300 and β-TrCP and promotes cancer malignancy. Nat Commun.

[113] Komura N (2020). The role of myeloid-derived suppressor cells in increasing cancer stem-like cells and promoting PD-L1 expression in epithelial ovarian cancer. Cancer Immunol Immunother.

[114] de Almeida Nagata DE (2019). Regulation of tumor-associated myeloid cell activity by CBP/EP300 bromodomain modulation of H3K27 acetylation. Cell Rep.

[115] Xiong J (2022). Lactylation-driven METTL3-mediated RNA m^6^A modification promotes immunosuppression of tumor-infiltrating myeloid cells. Mol Cell.

[116] Lu Z (2020). Epigenetic therapy inhibits metastases by disrupting premetastatic niches. Nature.

[117] Haverkamp JM (2014). Myeloid-derived suppressor activity is mediated by monocytic lineages maintained by continuous inhibition of extrinsic and intrinsic death pathways. Immunity.

[118] Mikyšková R (2014). DNA demethylating agent 5-azacytidine inhibits myeloid-derived suppressor cells induced by tumor growth and cyclophosphamide treatment. J Leukoc Biol.

[119] Shang W (2019). The pseudogene *Olfr29-ps1* promotes the suppressive function and differentiation of monocytic MDSCs. Cancer Immunol Res.

[120] Shaul ME, Fridlender ZG (2019). Tumour-associated neutrophils in patients with cancer. Nat Rev Clin Oncol.

[121] Veglia F (2021). Myeloid-derived suppressor cells in the era of increasing myeloid cell diversity. Nat Rev Immunol.

[122] Luo S (2023). METTL3-mediated m6A mRNA methylation regulates neutrophil activation through targeting TLR4 signaling. Cell Rep.

[123] Hamam HJ (2019). Histone acetylation promotes neutrophil extracellular trap formation. Biomolecules.

[124] Goswami S (2023). Myeloid-specific KDM6B inhibition sensitizes glioblastoma to PD1 blockade. Nat Cancer.

[125] Hedrick CC, Malanchi I (2021). Neutrophils in cancer: heterogeneous and multifaceted. Nat Rev Immunol.

[126] Kalafati L (2020). Innate immune training of granulopoiesis promotes anti-tumor activity. Cell.

[127] Chen Y-P (2021). Unraveling tumour microenvironment heterogeneity in nasopharyngeal carcinoma identifies biologically distinct immune subtypes predicting prognosis and immunotherapy responses. Mol Cancer.

[128] Ziogas DC (2023). Mechanisms of resistance to immune checkpoint inhibitors in melanoma: What we have to overcome?. Cancer Treat Rev.

[129] Zeng D (2021). Tumor microenvironment evaluation promotes precise checkpoint immunotherapy of advanced gastric cancer. J Immunother Cancer.

[130] Liu B (2022). BRD4-directed super-enhancer organization of transcription repression programs links to chemotherapeutic efficacy in breast cancer. Proc Natl Acad Sci U S A.

[131] Hogg SJ (2020). Targeting the epigenetic regulation of antitumour immunity. Nat Rev Drug Discov.

[132] Anichini A (2022). Landscape of immune-related signatures induced by targeting of different epigenetic regulators in melanoma: implications for immunotherapy. J Exp Clin Cancer Res.

[133] Fu Y (2022). The DNMT1-PAS1-PH20 axis drives breast cancer growth and metastasis. Signal Transduct Target Ther.

[134] Wang X (2020). HDAC inhibitors overcome immunotherapy resistance in B-cell lymphoma. Protein Cell.

[135] Dhillon S (2020). Decitabine/cedazuridine: first approval. Drugs.

[136] Gonda TA (2020). A DNA hypomethylating drug alters the tumor microenvironment and improves the effectiveness of immune checkpoint inhibitors in a mouse model of pancreatic cancer. Cancer Res.

[137] Kim H (2020). PRMT5 control of cGAS/STING and NLRC5 pathways defines melanoma response to antitumor immunity. Sci Transl Med.

[138] Lv J (2023). Epigenetic modification of *CSDE1* locus dictates immune recognition of nascent tumorigenic cells. Sci Transl Med.

[139] Nigam N (2023). SMYD3 represses tumor-intrinsic interferon response in HPV-negative squamous cell carcinoma of the head and neck. Cell Rep.

[140] Hu R (2021). PRMT5 inhibition promotes PD-L1 expression and immuno-resistance in lung cancer. Front Immunol.

[141] Krekorian M (2019). Imaging of T-cells and their responses during anti-cancer immunotherapy. Theranostics.

[142] Liu Y (2021). LSD1 inhibition sustains T cell invigoration with a durable response to PD-1 blockade. Nat Commun.

[143] Micevic G (2023). IL-7R licenses a population of epigenetically poised memory CD8^+^ T cells with superior antitumor efficacy that are critical for melanoma memory. Proc Natl Acad Sci U S A.

[144] Wu B (2022). RNA polymerase II pausing factor NELF in CD8^+^ T cells promotes antitumor immunity. Nat Commun.

[145] Itahashi K (2022). BATF epigenetically and transcriptionally controls the activation program of regulatory T cells in human tumors. Sci Immunol.

[146] Chen Z (2023). A new histone deacetylase inhibitor remodels the tumor microenvironment by deletion of polymorphonuclear myeloid-derived suppressor cells and sensitizes prostate cancer to immunotherapy. BMC Med.

[147] Zhang X (2012). SAHA, an HDAC inhibitor, synergizes with tacrolimus to prevent murine cardiac allograft rejection. Cell Mol Immunol.

[148] Pappalardi MB (2021). Discovery of a first-in-class reversible DNMT1-selective inhibitor with improved tolerability and efficacy in acute myeloid leukemia. Nat Cancer.

[149] Oki Y (2008). Induction of hypomethylation and molecular response after decitabine therapy in patients with chronic myelomonocytic leukemia. Blood.

[150] Scheller M (2021). Hotspot DNMT3A mutations in clonal hematopoiesis and acute myeloid leukemia sensitize cells to azacytidine via viral mimicry response. Nat Cancer.

[151] Khateb A (2021). The ubiquitin ligase RNF5 determines acute myeloid leukemia growth and susceptibility to histone deacetylase inhibitors. Nat Commun.

[153] El Khawanky N (2021). Demethylating therapy increases anti-CD123 CAR T cell cytotoxicity against acute myeloid leukemia. Nat Commun.

[154] Zhang Y, Zhang Z (2020). The history and advances in cancer immunotherapy: understanding the characteristics of tumor-infiltrating immune cells and their therapeutic implications. Cell Mol Immunol.

[155] Morad G (2021). Hallmarks of response, resistance, and toxicity to immune checkpoint blockade. Cell.

[156] Reardon DA (2020). Effect of nivolumab vs bevacizumab in patients with recurrent glioblastoma: The CheckMate 143 phase 3 randomized clinical trial. JAMA Oncol.

[157] Miguel M de, Calvo E (2020). Clinical challenges of immune checkpoint inhibitors. Cancer Cell.

[158] Lim SY (2023). The molecular and functional landscape of resistance to immune checkpoint blockade in melanoma. Nat Commun.

[159] Tang T (2021). Advantages of targeting the tumor immune microenvironment over blocking immune checkpoint in cancer immunotherapy. Signal Transduct Target Ther.

[160] Khan F (2023). Macrophages and microglia in glioblastoma: heterogeneity, plasticity, and therapy. J Clin Invest.

[161] Yang J (2023). Epigenetic regulation in the tumor microenvironment: molecular mechanisms and therapeutic targets. Signal Transduct Target Ther.

[162] Truong AS (2021). Entinostat induces antitumor immune responses through immune editing of tumor neoantigens. J Clin Invest.

[163] Vogelstein B (2013). Cancer genome landscapes. Science.

[164] Easwaran H (2014). Cancer epigenetics: tumor heterogeneity, plasticity of stem-like states, and drug resistance. Mol Cell.

[165] Sundar R (2022). Epigenetic promoter alterations in GI tumour immune-editing and resistance to immune checkpoint inhibition. Gut.

[166] Souroullas GP (2016). An oncogenic Ezh2 mutation induces tumors through global redistribution of histone 3 lysine 27 trimethylation. Nat Med.

[167] Soriano-Baguet L, Brenner D (2023). Metabolism and epigenetics at the heart of T cell function. Trends Immunol.

[168] Amit I (2016). The role of the local environment and epigenetics in shaping macrophage identity and their effect on tissue homeostasis. Nat Immunol.

[169] Liu P-S (2023). CD40 signal rewires fatty acid and glutamine metabolism for stimulating macrophage anti-tumorigenic functions. Nat Immunol.

[170] Lauterbach MA (2019). Toll-like receptor signaling rewires macrophage metabolism and promotes histone acetylation via ATP-Citrate lyase. Immunity.

[171] Zhang M (2021). Pancreatic cancer cells render tumor-associated macrophages metabolically reprogrammed by a GARP and DNA methylation-mediated mechanism. Signal Transduct Target Ther.

[172] Wu SJ (2021). Single-cell CUT&Tag analysis of chromatin modifications in differentiation and tumor progression. Nat Biotechnol.

[173] Lucas MC, Novoa EM (2023). Long-read sequencing in the era of epigenomics and epitranscriptomics. Nat Methods.

[174] Sen DR (2016). The epigenetic landscape of T cell exhaustion. Science.

[175] Lin D (2022). Decoding the spatial chromatin organization and dynamic epigenetic landscapes of macrophage cells during differentiation and immune activation. Nat Commun.

[176] Zhang P (2022). Epigenomic analysis reveals a dynamic and context-specific macrophage enhancer landscape associated with innate immune activation and tolerance. Genome Biol.

